# Antibiotic exposure exacerbates acute-on-chronic liver failure via gut microbiota imbalance and secondary liver lesion

**DOI:** 10.1099/jmm.0.002045

**Published:** 2026-01-22

**Authors:** Shujuan Yang, Jing Wang, Nan Yang, Juan Li, Li Jin, Yan Zhang, Hongli Wang, JianJun Fu, Tianyan Chen, Yingren Zhao, Yingli He

**Affiliations:** 1 Department of Infectious Diseases, The First Affiliated Hospital of Xi’an Jiaotong University, Xi’an 710061, Shaanxi, PR China; 2 Department of Infectious Diseases, The Eighth Hospital of Xi’an, Xi’an 710061, Shaanxi, PR China; 3 Institution of Hepatology, The First Affiliated Hospital of Xi’an Jiaotong University, Xi’an 710061, Shaanxi, PR China; 4 Clinical Research Center for Infectious Diseases of Shaanxi, Xi’an 710061, Shaanxi, PR China; 5 Department of Rheumatology, The First Affiliated Hospital of Xi’an Jiaotong University, Xi’an 710061, Shaanxi, PR China; 6 Department of Infectious Diseases, Xi’an Central Hospital, Xi’an 710061, Shaanxi, PR China

**Keywords:** acute-on-chronic liver failure, antibiotic exposure, dysbiosis, gut–liver axis, inflammation, microbiota

## Abstract

**Introduction.** The correlation between antibiotic exposure and adverse outcomes in patients with acute-on-chronic liver failure (ACLF) remains controversial, and the underlying mechanism is unclear.

**Hypothesis/Gap Statement.** This study hypothesizes that antibiotic exposure in ACLF patients alters gut microbiota, which affects the outcome of ACLF.

**Aim.** To explore the effect of antibiotic exposure on gut microbiota that affects the outcome of ACLF.

**Methodology.** A retrospective matched study of ACLF patients and the ACLF rat model was used to assess adverse outcomes associated with antibiotic exposure. The gut microbiota of the ACLF patients and the ACLF rat model were sequenced using the Illumina MiSeq platform.

**Results.** Twenty-three ACLF patients who were exposed to antibiotics and 46 matched controls who were not exposed to antibiotics were enrolled. The survival rates at 4, 12 and 24 weeks were significantly lower in the exposure group than in the non-exposure group. In the ACLF rat model, hepatitis in the antibiotic-exposure group became more severe, and the alanine transaminase levels were higher than those of the non-exposure group. The gut microbiota diversity was decreased in the ACLF patients with antibiotic exposure, and the proportions of *Enterococcaceae* and *Peptostreptococcaceae* were increased, while those of *Lachnospiraceae*, *Bifidobacteriaceae* and *Bacteroidaceae* were decreased. In the rat model, antibiotic exposure induced Gram-positive and Gram-negative bacterial eradication, and *Klebsiella* became the dominant micro-organism.

**Conclusion.** Antibiotic exposure aggravated hepatitis and had no survival benefit for ACLF. The underlying mechanism may be related to dysbiosis in the gut microbiota.

## Data Summary

The datasets presented in this study can be found in online repositories. The names of the repository/repositories and accession number(s) can be found below: the NCBI Sequence Read Archive database (http://www.ncbi.nlm.nih.gov/bioproject), and the BioProject number is PRJNA1068357 and individual accession numbers can be found in Table S1 (available in the online Supplementary Material).

## Introduction

Acute-on-chronic liver failure (ACLF) is a clinical syndrome that is characterized by acute and severe hepatic abnormalities in patients with pre-existing chronic liver disease or cirrhosis as well as high 28-day mortality rates of 50–90% [1]. Bacterial infections are a main precipitant or a common complication and increase the ACLF-associated mortality rates [2,3]. Approximately 58% of ACLF patients are reported to experience bacterial infections during hospitalization [4]. The previous study showed that these patients exhibited higher 90-day mortality rates compared with those without infections (51% vs. 38%) [5]. Empiric antibiotic prophylaxis was previously thought to reduce mortality among ACLF patients [6]. However, Karvellas *et al*. reported that antibiotic prophylaxis did not decrease the incidence of bloodstream infections, liver transplants or overall mortality within 21 days of its administration [7]. Habib *et al*. performed a retrospective study with a large sample size of 457 ACLF patients and demonstrated that antibiotic treatment did not improve patient survival [8], which gradually changed the belief regarding whether antibiotic prophylaxis could benefit ACLF patients. However, in that study, the main cirrhosis type was hepatitis C virus-induced, which is not the main cause of cirrhosis in Asian patients, and confounding factors such as age, sex and disease severity may have affected the study results. Second, antibiotic treatment caused short-term adverse effects, including diarrhoea, *Clostridium difficile* infection and selection of antibiotic-resistant micro-organisms. Third, several antibiotics are hepatotoxic or nephrotoxic, and some can lead to cholestasis or further complications for ACLF patients [9,10]. Based on the cost–benefit ratio, antibiotic prophylaxis is no longer recommended for ACLF patients [7,8, 11].

The gut–liver axis, in which the hepatic portal system transports blood derived from the gut, accounts for the ACLF pathogenesis [12,13]. When the gut barriers, including the mucosal, microbiota and peristalsis barriers, become impaired, the prognosis for ACLF patients sharply deteriorates. Among these barriers, the microbiota barrier plays a vital role in blocking harmful substances from reaching the liver. The gut microbiota in the gastrointestinal tract influences liver metabolism via nutrient absorption and bile acid homeostasis [14,15] and regulates systemic inflammation and disease severity via bacteria and bacterial products [16,17]. However, no adequate evidence exists to support a correlation between severe hepatitis in patients with ACLF and the gut microbiota.

Herein, we used the ACLF rat model to evaluate the effect of antibiotics on hepatic inflammation. We investigated the correlation between hepatic inflammation and microbiota barriers in ACLF. A retrospective matched study of ACLF patients with hepatitis B virus-induced cirrhosis was performed to confirm this correlation and the adverse outcomes due to ACLF.

## Methods

### Patients

One hundred and forty-four patients with ACLF were included at the First Affiliated Hospital of Xi’an Jiaotong University from 2017 to 2019. Patients were included if they met the diagnostic criteria for ACLF in line with the 2014 Asia-Pacific Liver Disease Association [18] and had no fever, a white blood cell (WBC) count of <10.0×10^9^ l^−1^, procalcitonin (PCT) of <0.5 ng l^−1^, and no signs of infection on chest computed tomography (CT). Patients were excluded if they were taking antibiotic medications, diagnosed as enteritis, hypertension, diabetes, autoimmune disease or cancer or coinfected with hepatitis A, C, D, E and human immunodeficiency virus.

**Fig. 1.**
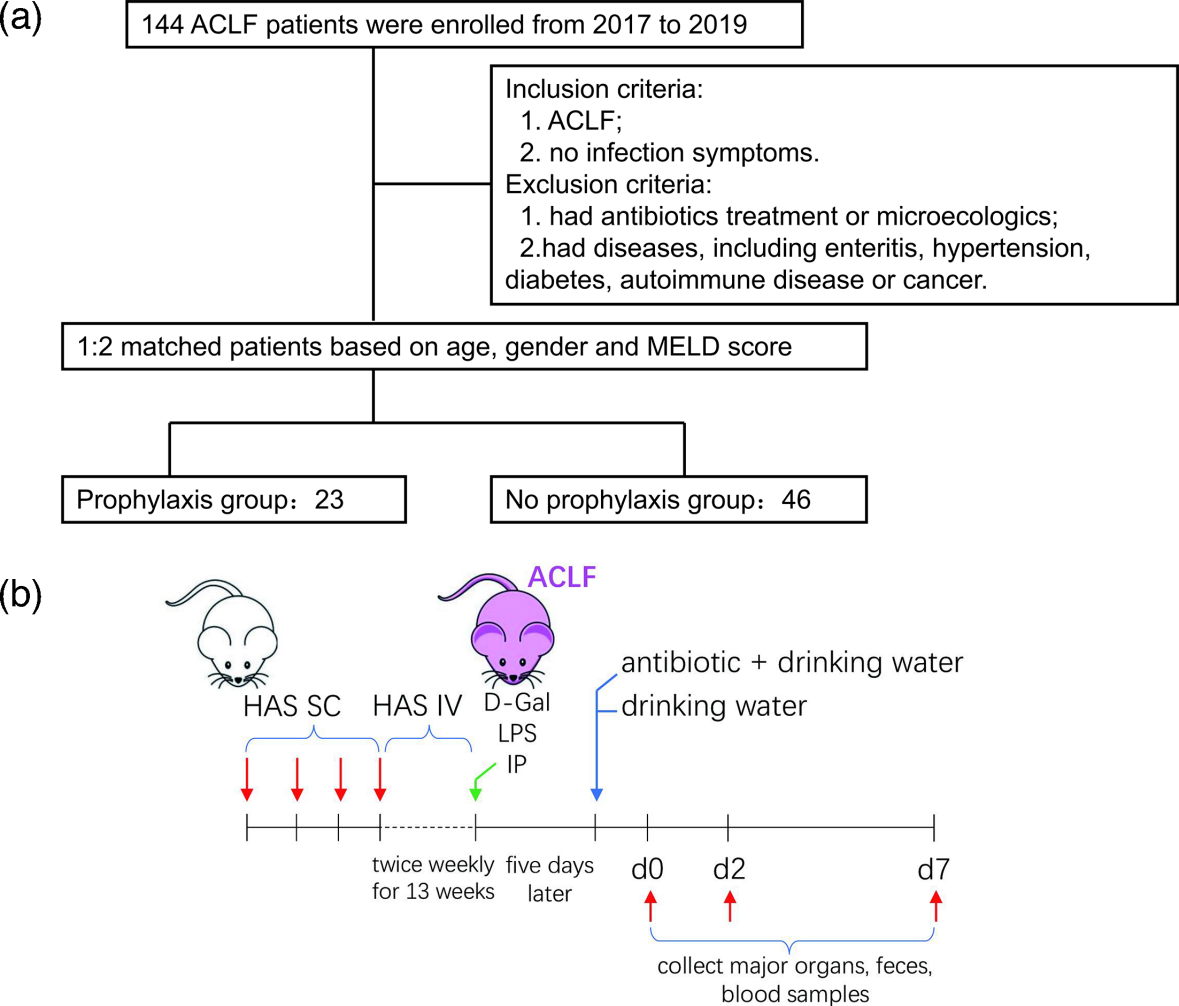
Flowchart of the study. (**a**) Flowchart of ACLF patient matching. (**b**) Flowchart of the ACLF rat model.

Patients who were undergoing antibiotic treatment and patients who were age-, sex- and model for end-stage liver disease (MELD) score-matched without antibiotic exposure (a 1 : 2 ratio) were included in this retrospective matched study. Patients receiving antibiotic treatment were defined as the antibiotic group (exposure group), and the age-, sex- and MELD score-matched patients without antibiotic exposure were included in the control group (no exposure group, Fig. 1b). In this study, we used the actual sample size and the calculated effect size to give a power of 0.80. Detailed information, including demographics, laboratory tests and final clinical outcomes, was collected from the recruited patients’ medical records or telephone follow-up records. The primary outcome was the 28-day survival rate. The secondary outcomes were the 12-week and 24-week survival rates and changes in the gut microbiota.

The alanine transaminase (ALT), total bilirubin, total cholesterol, serum albumin, fasting glucose, serum creatinine, haemoglobin, WBC count, platelet, prothrombin time, international normalized ratio (INR) and PCT tests were performed in the central laboratory at the First Affiliated Hospital of Xi’an Jiaotong University.

### Animals and antibiotic treatment

Sprague–Dawley male Wistar rats (100–130 g) were obtained from the Animal Center of Xi’an Jiaotong University, maintained under a specific pathogen-free facility and cared for in accordance with the Guide for the Care and Use of Laboratory Animals (1996). All experiments were performed in accordance with protocols approved by the Institutional Animal Care and Use Committee of Xi’an Jiaotong University.

The schematic of the ACLF animal model and antibiotic treatment is shown in Fig. 1a. The immune-mediated chronic liver failure was induced by human serum albumin (HSA) as previously described. HSA (Octapharma GmbH, Austria) was diluted in saline and emulsified with Freund’s incomplete adjuvant. Each rat received 4 mg HSA with each injection. This was done subcutaneously with a 14-day interval for the first four infections, followed by tail vein injection twice weekly for another 13 weeks. Acute endotoxemia induced by intravenous administration of d-galactosamine (d-Gal) (Sigma-Aldrich) and lipopolysaccharide (LPS) (Sigma-Aldrich) was used to cause an acute deterioration of cirrhosis, mimicking ACLF. The animals were treated with d-Gal (400 mg kg^−1^) and LPS (100 µg kg^−1^). Antibiotic treatment started 5 days later, after d-Gal and LPS administration was stopped.

Rats, randomly divided into groups of twenty each, were given combined antibiotics (500 mg l^−1^ vancomycin, 1 g l^−1^ neomycin, 1 g l^−1^ ampicillin and 1 g l^−1^ metronidazole) in drinking water. Control rats received drinking water only. We maintained another ten rats without ACLF-induced toxicant administration as the normal group. The day after the antibiotic treatment was defined as baseline. At baseline, day 2 and day 7, rats were anesthetized and sacrificed with pentobarbital. Major organs and faeces were collected and then stored at −80 ℃ for further analysis. Sera obtained from blood samples was stored in small aliquots at −80 ℃ until further analysis. Cross-sections of the liver were fixed in 10% buffered formalin and stained using haematoxylin and eosin. The degree of inflammatory activity (G) was used to quantify the histological inflammation in the liver tissue.

### Microbiome analysis (16S rRNA sequencing)

Taking faecal samples from the cecum of antibiotic-exposed rats and controls, DNA isolation and 16S rRNA sequencing were performed. Briefly, total bacterial genomic DNA was extracted using the QIAamp DNA Stool Mini Kit (QIAGEN, Hilden, Germany). The 16S v3~v4 region was amplified by PCR and sequenced on the MiSeq platform (Illumina, San Diego, CA) using a 2×250 bp paired-end protocol, yielding paired-end reads that overlap almost completely. Agile Toolkit for Incisive Microbial Analyses (ATIMA) was used to analyse microbiome data, which is a stand-alone tool for analysing and visualizing trends in taxa abundance, *α*-diversity and *β*-diversity as they relate to sample metadata. Quantitative PCR was performed to further verify certain bacterial changes.

### Statistical analysis

Continuous variables are presented as the mean±sd; categorical variables are presented as the count (percentage). The *χ*2 test or Fisher’s exact test was used to compare two groups. The Shapiro–Wilk test and Levene statistic were used to evaluate the normality and homogeneity of the variance, respectively. Differences between the two groups were evaluated using t-tests or the Mann–Whitney *U* test. Adjustment for multiple comparisons was performed using Student–Newman–Keuls (S-N-K). The Cox regression test was used to analyse the effect of antibiotic prophylaxis on the survival rate. All tests were two-sided, and *P* values<0.05 were statistically significant. All analyses were performed using SPSS software v. 24.0 (IBM Corp., Armonk, NY, USA).

## Results

### Baseline characteristics of the study cohort

We consecutively enrolled 23 ACLF patients with antibiotic exposure. To adjust for the effect of age, sex and disease severity on gut microbiota composition, we selected 46 unexposed ACLF patients from our database matched for age, sex and MELD score as the control. The clinical characteristics of all patients are presented in Table 1. There was no significant difference between patients with antibiotic exposure group patients and controls in terms of ALT, total bilirubin, INR, total cholesterol, plasma ammonia, serum albumin, fasting glucose, serum creatinine, WBC, platelets, haemoglobin and PCT at the baseline.

**Table 1.** Baseline characteristics of the ACLF patients

Variable	No exposure group (*N*=46)	Exposure group (*N*=23)	*P* value
Age, years	43.4±12.6	42.8±15.4	0.866
Gender, male (%)	34 (73.9%)	17 (73.9%)	1.000
MELD score	21.1 (16.8–51.5)	20.5 (15.5–36.0)	0.674
ALT, U l^−1*^	292.0 (21.2–4690.0)	359.1 (33.0–2954.2)	0.346
Total bilirubin, umol l^−1*^	218.1 (108.7–535.0)	299.3 (125.2–562.6)	0.103
INR^*^	1.8 (1.5–16.5)	1.9(1.5–5.5)	0.380
Total cholesterol, mmol l^−1^	2.0±0.7	1.8±0.8	0.287
Plasma ammonia, umol l^−1^	89.7±59.7	104.9±58.2	0.571
Serum albumin, g l^−1^	31.7±4.6	30.0±3.4	0.111
Fasting glucose, mmol l^−1*^	4.6 (2.9–6.9)	4.6 (3.5–6.4)	0.891
Serum creatinine, umol l^−1*^	72.8 (40.0–404.6)	67.4 (43.0–115.6)	0.652
WBC, 109 l^−1^	4.6±2.1	4.8±1.3	0.634
PLT, 109 l^−1*^	94.5 (25.0–214.0)	87.0 (40.0–218.0)	0.597
HGB, g l^−1^	123.3±20.3	125.7±16.8	0.624
PCT, ng l^−1^	0.02±0.02	0.06±0.03	0.087

*Values are expressed as the median (range), others are expressed as the mean±sd.

HGB, haemoglobin; PLT, platelets.

### Antibiotic exposure exacerbates ACLF patients’ survival

**Fig. 2.**
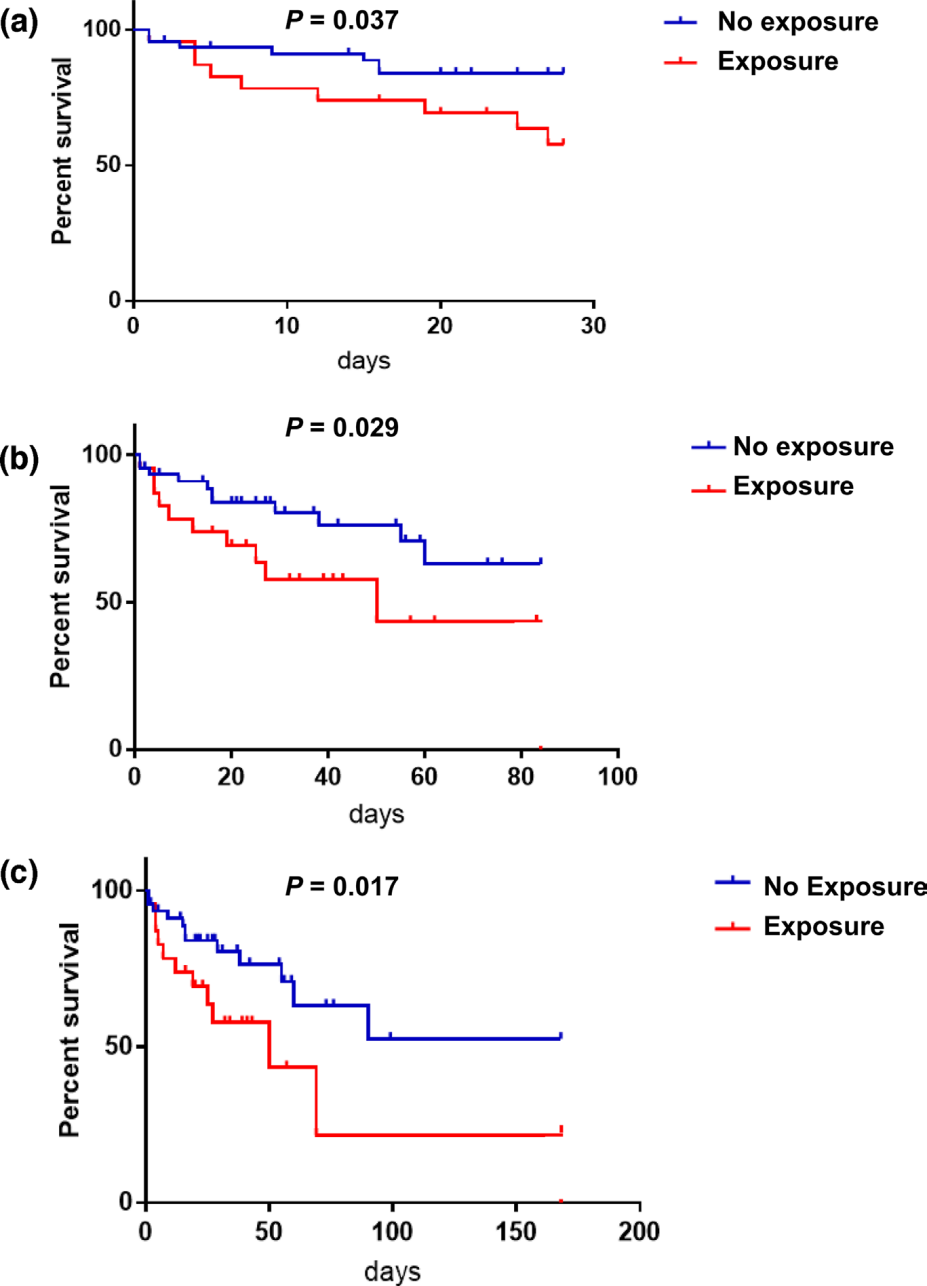
Kaplan–Meier curves showing the effect of antibiotic exposure on survival time in ACLF patients. (**a**) Effect of antibiotic exposure on 4-week survival. (**b**) Effect of antibiotic exposure on 12-week survival. (**c**) Effect of antibiotic exposure on 24-week survival.

The survival of antibiotic-exposed patients was significantly lower than unexposed patients (*P*=0.029 at 12 weeks and *P*=0.017 at 24 weeks, Fig. 2b, c), and antibiotic-exposed patients showed significantly higher early-mortality rate than control (*P*=0.037 at 28 days, Fig. 2a). The antibiotic-exposed cohort also showed a bit higher total mortality rate (47.8%, 11/23) than control (28.3%, 13/46), although the difference was not significant (*χ2*=2.588, *P*=0.108).

### Antibiotic exposure induces hepatic inflammatory responses in ACLF rats

To demonstrate that, the ACLF rat model was confirmed by the histological assessment of the liver. ACLF was represented by congestion, haemorrhages, multifocal to coalescing areas of coagulative necrosis, randomly distributed within the hepatic lobules or centred on periportal regions and associated with severe and mixed inflammatory infiltrates. The portal spaces were also affected and expanded by fibrosis, bile duct hyperplasia and large numbers of inflammatory cells, predominated by small lymphocytes and macrophages (Fig. 1a). There were no significant structural changes in normal groups (Fig. 1a).

**Fig. 3.**
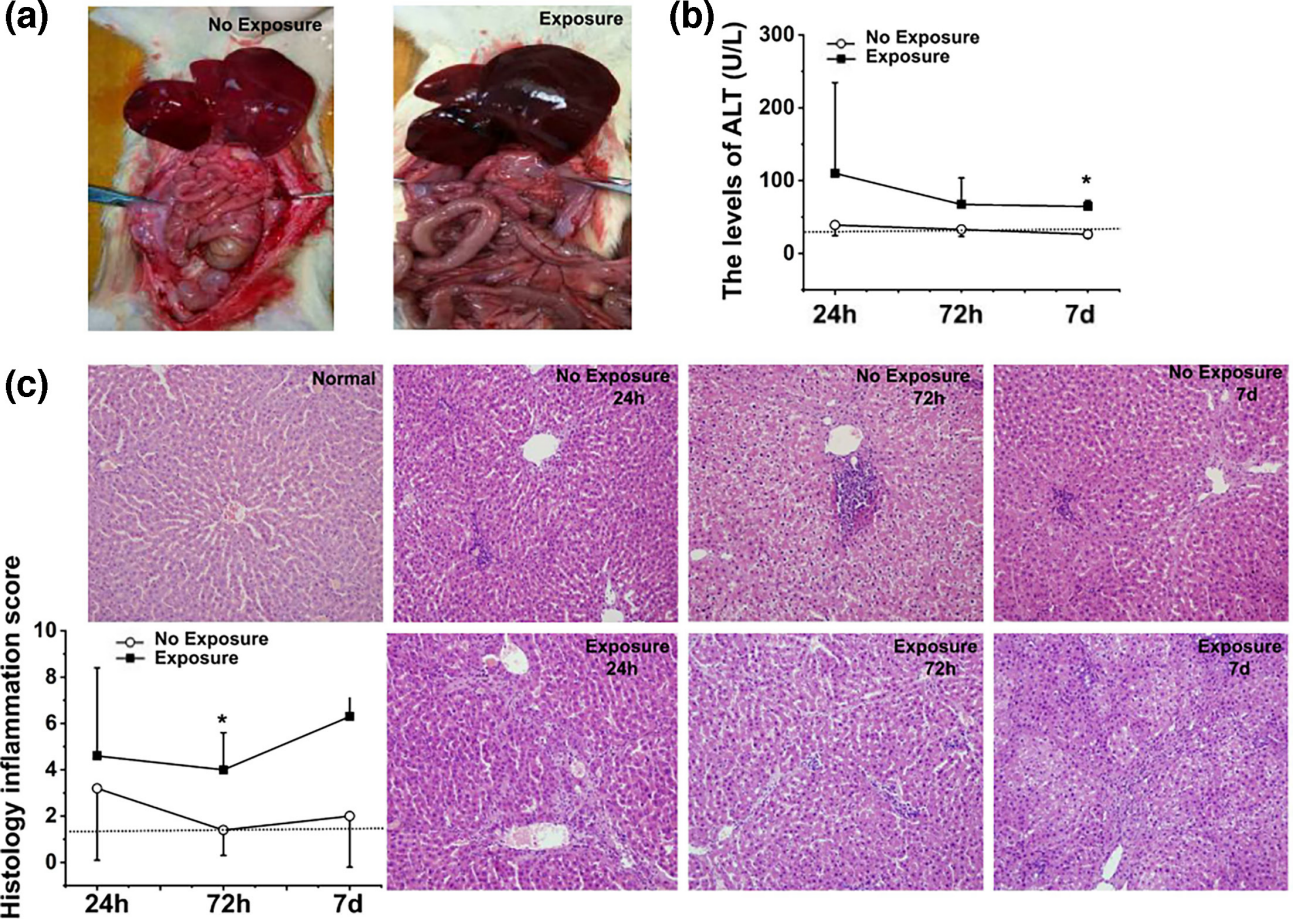
Hepatic flare in ACLF model rats. (**a**) Morphology of the exposure and non-exposure groups. (**b**) ALT in the exposure and non-exposure groups. The dotted lines in each graph show the ALT (34.0±7.2 U l^−1^) levels in the normal controls. (**c**) Haematoxylin and eosin staining and histological scores in the exposure and non-exposure groups.

Antibiotic treatment led to congestion and oedema in the liver and intestines (Fig. 3a). In addition, antibiotic exposure led to a significant increase in ALT (*P*＜0.05, Fig. 3b), indicating that antibiotic treatment exacerbates the hepatic inflammatory activities. Moreover, increased infiltration of immune cells and histological inflammation score were also found in the exposed cohort (*P* < 0.05, Fig. 3c). The observed increases in ALT, along with histological inflammation score, suggest the onset of hepatitis in ACLF rats after antibiotic exposure.

### Antibiotic exposure alters gut microbiome composition in ACLF rats

**Fig. 4.**
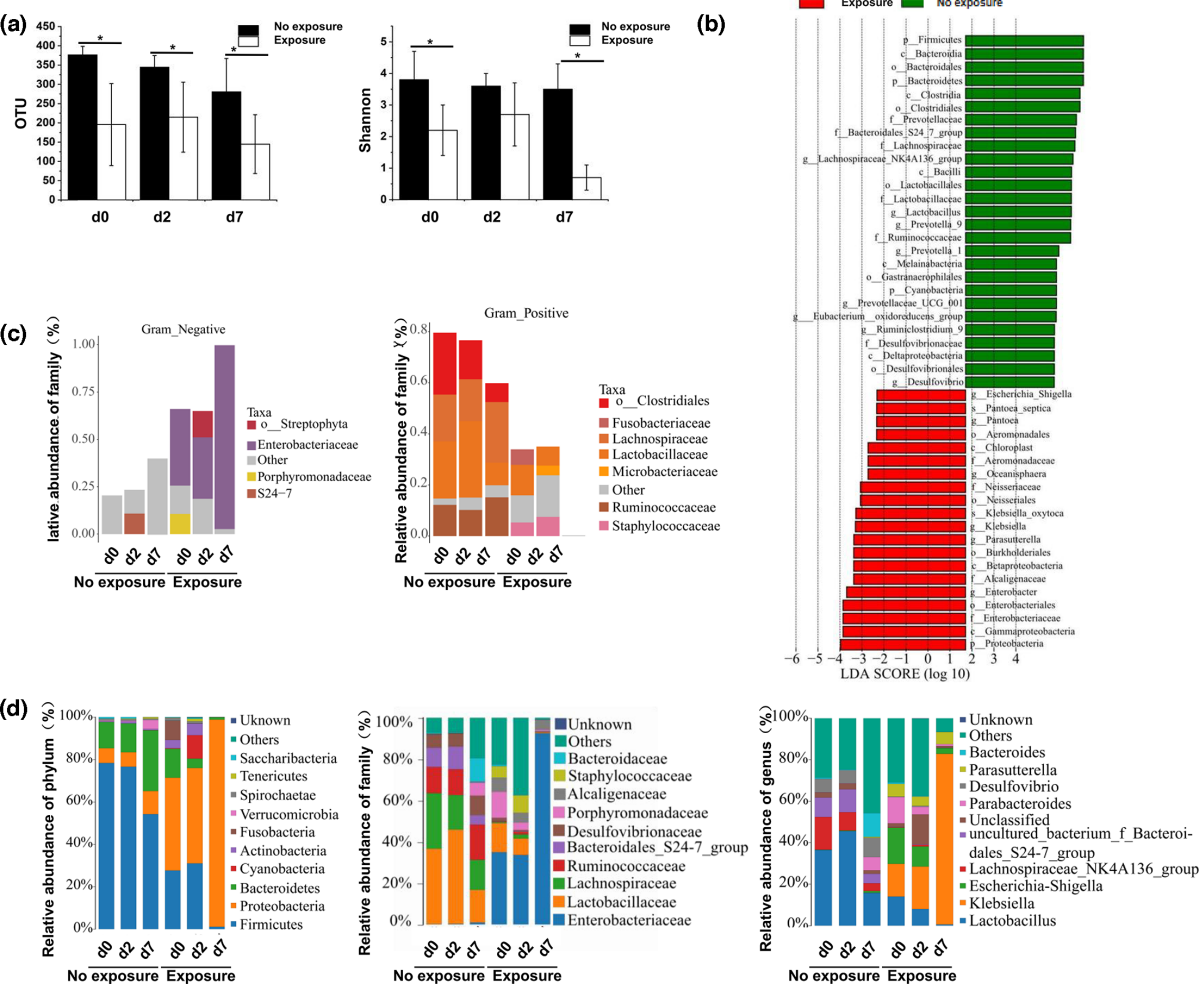
Gut microbiota disruptions due to antibiotic exposure in ACLF model rats. (**a**) Comparison of the microbial diversity exposure and non-exposure groups. (**b**) The LDA score identified the size differentiation between the exposure and non-exposure groups with a threshold value of 3.5. (**c**) Comparison of Gram-positive and Gram-negative bacterial distributions between the exposure and non-exposure groups. (**d**) Comparison of the microbiota distribution at the phylum, family and genus levels between the exposure and non-exposure groups.

Since the intestinal congestion and oedema were observed in antibiotic-exposed rats, we speculated that the gut barriers were impaired. To determine the potential of antibiotics in causing impaired microbiota barriers, faecal samples from antibiotic-treated and control rats were collected at d0, d2 and d7 and subjected to 16S rRNA sequencing. The *α*-diversity of bacterial communities was evaluated according to observed operational taxonomic units (OTUs) and Shannon’s diversity index. Antibiotic exposure led to a significant decrease in OTUs and Shannon’s diversity index (Fig. 4a), indicating that antibiotic treatment reduced the microbial diversity. As revealed by *β*-diversity analysis, antibiotic exposure resulted in distinct bacterial community clustering (Fig. 4b). Notably, differences were observed for both unweighted and weighted measures, indicating that antibiotic treatment impacted not only the type of bacteria present but also the distribution of shared bacteria.

We subsequently identified significant antibiotic-induced changes at different taxonomical levels, including 12 bacterial phyla, 12 bacterial families and 12 bacterial genera. Linear discriminant analysis effect size (LEfSe) analysis identified the high-dimensional differences in the gut microbiota distributions between the exposure and no exposure groups (Fig. 4b). BugBase analysis showed that the Gram-positive (G+) bacteria were reduced in both groups, Gram-negative (G–) bacteria were eliminated and *Enterobacteriaceae* became the dominant G– bacteria in the exposure group (Fig. 4c).

Specifically, we observed reductions in the relative abundance of bacterial families *Lactobacillaceae* and *Lachnospiraceae* under *Firmicutes* phylum and enrichment in bacterial families *Enterobacteriaceae* under *Proteobacteria* phylum in antibiotic-treated animals as compared to controls (Fig. 4d). Antibiotic exposure led to reductions at the genus level including *Lactobacillus* and *Lachnospira* under *Lactobacillaceae* and *Lachnospiraceae* families, respectively (Fig. 4d), which are established probiotics known to promote induction of Treg cells, and their reduced levels could thus promote intestinal inflammatory responses. On the other hand, antibiotic exposure also showed enrichment of *Klebsiella* and *Escherichia–Shigella* genera under the *Enterobacteriaceae* family (Fig. 4d), which are conditioned pathogens. Another decreasing trend in the *Bacteroidales* genus under the *Bacteroidetes* phylum was also observed in antibiotic-exposed animals (Fig. 4d). Our data thus show that antibiotic exposure causes significant alterations in the gut microbiome and suggest an association of gut dysbiosis with ACLF pathogenesis.

### Antibiotic exposure alters gut microbiome composition in ACLF patients

**Fig. 5.**
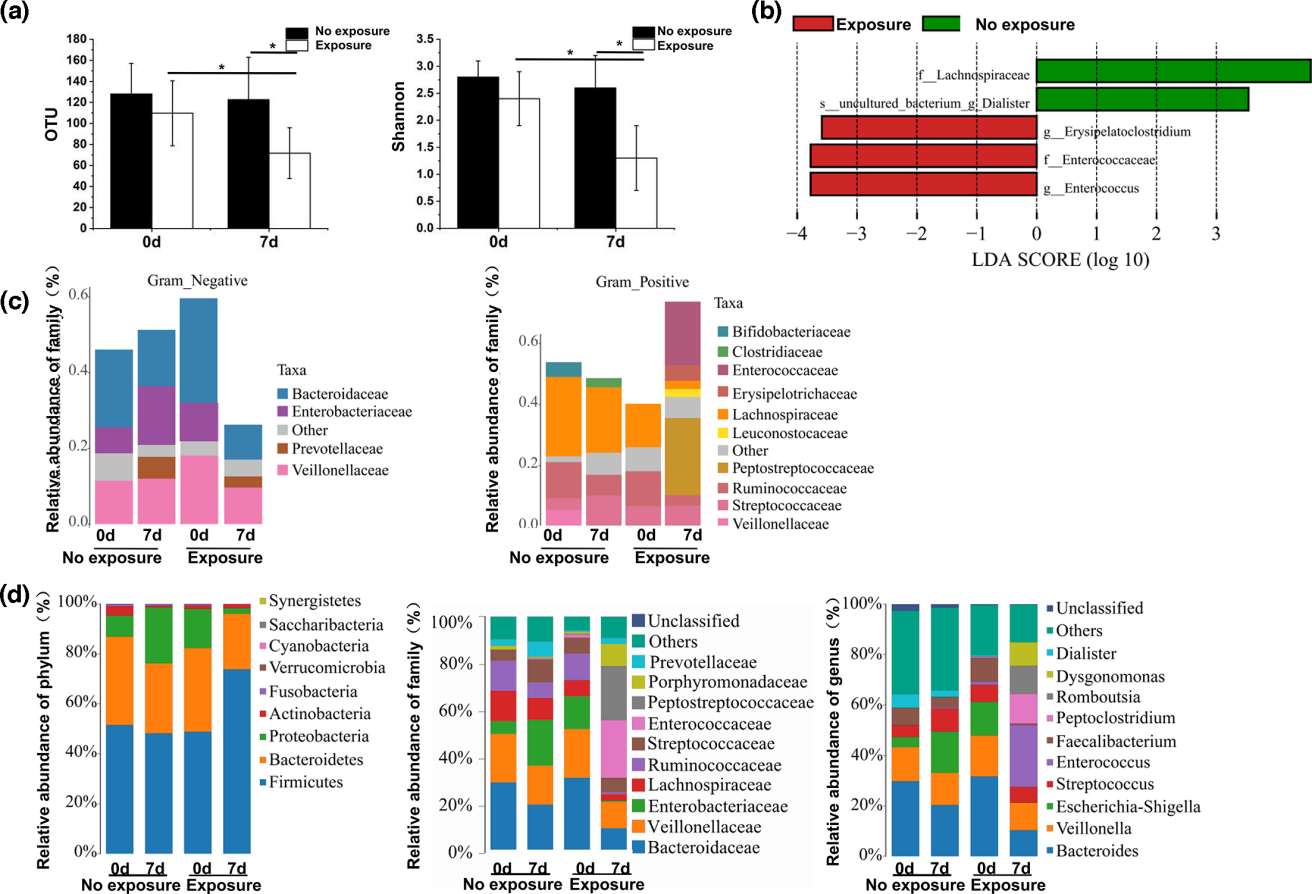
Gut microbiota disruptions due to antibiotic exposure in ACLF patients. (**a**) Comparison of the microbial diversity between the exposure and non-exposure groups. (**b**) LDA score differentiated between the sizes in the exposure and non-exposure groups with a threshold value of 3.5. (**c**) Comparison of Gram-positive and Gram-negative bacterial distributions between the exposure and non-exposure groups. (**d**) Comparison of the microbiota distribution at the phylum, family and genus levels between the exposure and non-exposure groups.

To confirm the differences in the gut microbiota in ACLF patients before and after antibiotic exposure, total genomic DNA was sequenced from their faecal samples. Consistent with the ACLF rats, the OTUs and Shannon values were lower in the exposure group than in the non-exposure group at d7 of antibiotic treatment (OTUs: 71.7±24.2 vs. 122.7±40.3, *t*=−2.871, *P*=0.014; Shannon value: 1.3±0.6 vs. 2.6±0.6, *t*=−3.697, *P*=0.003). Moreover, the OTUs and Shannon values were significantly decreased in the exposure group after 7 days of antibiotic exposure (OTUs: 109.7±30.9 vs. 71.7±24.2, *t*=2.559, *P*=0.025; Shannon value: 2.4±0.5 vs. 1.3±0.6, *t*=3.638, *P*=0.003). These values were not significantly decreased in the no exposure group (OTUs: 128.1±29.1 vs. 122.7±40.3, *t*=0.289, *P*=0.777; Shannon value: 2.8±0.3 vs. 2.6±0.6, *t*=0.783, *P*=0.449, Fig. 5a).

The microbiota proportion distribution changed after antibiotic exposure in the ACLF patients. LEfSe analysis showed that antibiotic exposure induced significant increases in *Erysipelatoclostridium*, *Enterococcaceae* and *Enterococcus* (common pathogens in ACLF patients) and significant decreases in *Lachnospiraceae* and *Dialister* in the exposure group (Fig. 5b). BugBase analysis was used to further compare the difference in G+ and G– bacteria. After 7 days of antibiotic exposure, the proportions of *Enterococcaceae* and *Peptostreptococcaceae* increased significantly for the G+ bacteria; additionally, the proportions of *Lachnospiraceae* and *Bifidobacteriaceae* (common probiotics) decreased for the G+ bacteria, and the proportions of *Bacteroidaceae* and *Enterobacteriaceae* decreased in the G– bacteria (Fig. 5c). However, the microbiota alterations were slight in the non-exposure group. Thus, antibiotic exposure induced gut microbiota perturbation at the phylum, family and genus levels (Fig. 5d). The proportions of some pathogens and conditional pathogens increased, the probiotic abundance decreased, and the proportion of *Enterococcus* increased in the exposure group after ACLF.

## Discussion

ACLF is characterized by high mortality, and there are few adequate therapies available in clinical practice. The benefit of antibiotic treatment remains controversial for ACLF patients. We found that antibiotic exposure induced gut microbiota perturbation, aggravated hepatic inflammation and increased mortality rates in patients with ACLF. To the best of our knowledge, these are the first results to suggest that antibiotic exposure negatively affects the outcomes of ACLF patients, which likely occurs by disturbing the gut microbiota.

Worldwide, antibiotic resistance is a continuing problem. The primary strategy to reduce the incidence of antibiotic resistance is rational antibiotic use. The rationalization of antibiotic treatment for ACLF patients remains controversial. Consistent with recent studies [6,8,19], our matched study, which controlled for confounding factors, confirmed that antibiotic use increased mortality rates in ACLF patients; however, the reason for this is unclear. Antibiotic overuse is causing an increase in antibiotic-resistant bacteria. An increase in antibiotic-resistant bacteria can lead to therapeutic difficulties and adverse outcomes for ACLF patients. However, antibiotic resistance does not occur in all patients. Other mechanisms also drive ACLF progression in antibiotic-exposed patients. Here, we used an ACLF rat model and found that broad-spectrum antibiotic exposure aggravated hepatic inflammation and elevated ALT levels. This is the first time that antibiotic use has been shown to exacerbate hepatic inflammation and contribute to adverse outcomes in ACLF.

The gut–liver axis plays a vital role in ACLF pathogenesis. The ACLF rat model showed that antibiotic-exposed rats had more swelling and congestion in their livers and guts compared with the no-exposure group. Antibiotics in this model exhibited no hepatotoxicity or absorbability. Thus, we speculated that the microbiota barrier destruction and increased pathogens were associated with adverse outcomes due to antibiotic exposure. First, the gut microbiota diversity was significantly reduced after antibiotic exposure in the ACLF rat model. The same result was confirmed in the ACLF patients; after 7 days of antibiotic treatment, the gut microbiota diversity notably decreased, whereas it did not significantly change in the unexposed patients. Second, the common probiotics, *Lachnospiraceae* and *Bifidobacteriaceae*, and the intestinal symbiotic bacteria, *Enterobacteriaceae*, decreased after antibiotic exposure. These alterations impaired the intestinal barrier and favoured bacteria and bacterial products that can produce inflammatory mediators and alter the bile acid composition. The pathogens and the products can then easily enter the blood, which may be associated with adverse outcomes in ACLF [20,22].

When the gut microbiota barrier is impaired, pathogen screening via antibiotics can cause a poor prognosis for patients with ACLF. In the ACLF rat model, broad-spectrum antibiotic exposure eradicated most bacteria, including both G+ and G– bacteria, and *Klebsiella* became the dominant bacterium. Notably, *Parasutterella* was present in the antibiotic-exposure rats; this differed from the non-exposure rats. *Parasutterella* is a relatively new G– bacterial genus from the *Proteobacteria* phylum, and studies on this bacterial genus are limited. Recent studies have associated an increased expression of *Parasutterella* with inflammatory bowel disease [23,24]. Antibiotic exposure increased the bacteria that are associated with gut inflammation, which may further impair the intestinal barrier. In ACLF patients, changes in the microbiota proportional distributions are complex because of the effects of diet and antibiotics. Various analyses consistently showed that certain pathogens and conditional pathogens (including *Enterococcaceae* and *Peptostreptococcaceae*) increased after 7 days of antibiotic exposure.

Here, we explored the association between the gut microbiota and antibiotic exposure in exacerbating ACLF. Although the study involving patients was retrospective and had a moderate sample size, the ACLF patients were matched by age, sex and MELD score, and important confounding factors were controlled. The gut microbiota is influenced by diet and antibiotic categories, which cannot be controlled in a retrospective patient study. To compensate for this, an ACLF rat model was used to show that hepatic inflammation was aggravated, and some pathogens became dominant after antibiotic exposure. The specific mechanisms by which dysbiosis worsens liver failure were not discussed in this study, which should be further investigated to explain the relationship between gut microbiota dysbiosis and the outcome of ACLF.

In conclusion, antibiotic exposure aggravated hepatic inflammation and did not improve the overall survival in the setting of ACLF. The mechanism by which antibiotic exposure induces adverse outcomes during ACLF may be associated with dysbiosis of the gut microbiota.

## Supplementary material

10.1099/jmm.0.002045 Uncited Table S1.

## Abbreviations

ACLF: acute-on-chronic liver failure
ALT: alanine transaminase
CT: computed tomography
d-Gal: d-galactosamine
HSA: human serum albumin
INR: international normalized ratio
LEfSe: linear discriminant analysis effect size
LPS: lipopolysaccharide
MELD: model for end-stage liver disease
OTUs: operational taxonomic units
PCT: procalcitonin
WBC: white blood cell
