# Supplementary material for: Antibiotic exposure exacerbates acute-on-chronic liver failure via gut microbiota imbalance and secondary liver lesion
Source: J Med Microbiol. 2026 Jan 22;75(1):002045. doi: 10.1099/jmm.0.002045 (PMC12825262; doi:10.1099/jmm.0.002045)
Supplement: Uncited Table S1. [file jmm-75-02045-s001.pdf]

sample_name	library_ID	sample accession numbers	title	library_strategy	library_source	library_selection
M1M_1	M1M_1	SAMN39586650	Effects of antibiotic to ACLF	CLONE	METAGENOMIC	PCR
M1M_2	M1M_2	SAMN39586651	Effects of antibiotic to ACLF	CLONE	METAGENOMIC	PCR
M2M_1	M2M_1	SAMN39586652	Effects of antibiotic to ACLF	CLONE	METAGENOMIC	PCR
M2M_2	M2M_2	SAMN39586653	Effects of antibiotic to ACLF	CLONE	METAGENOMIC	PCR
M3M_1	M3M_1	SAMN39586654	Effects of antibiotic to ACLF	CLONE	METAGENOMIC	PCR
M3M_2	M3M_2	SAMN39586655	Effects of antibiotic to ACLF	CLONE	METAGENOMIC	PCR
M4M_1	M4M_1	SAMN39586656	Effects of antibiotic to ACLF	CLONE	METAGENOMIC	PCR
M4M_2	M4M_2	SAMN39586657	Effects of antibiotic to ACLF	CLONE	METAGENOMIC	pCR
M7M_1	M7M_1	SAMN39586658	Effects of antibiotic to ACLF	CLONE	METAGENOMIC	PCR
M7M_2	M7M_2	SAMN39586659	Effects of antibiotic to ACLF	CLONE	METAGENOMIC	PCR
M8M_1	M8M_1	SAMN39586660	Effects of antibiotic to ACLF	CLONE	METAGENOMIC	PCR
M8M_2	M8M_2	SAMN39586661	Effects of antibiotic to ACLF	CLONE	METAGENOMIC	PCR
M11M_1	M11M_1	SAMN39586662	Effects of antibiotic to ACLF	CLONE	METAGENOMIC	PCR
M11M_2	M11M_2	SAMN39586663	Effects of antibiotic to ACLF	CLONE	METAGENOMIC	PCR
M12M_1	M12M_1	SAMN39586664	Effects of antibiotic to ACLF	CLONE	METAGENOMIC	PCR
M12M_2	M12M_2	SAMN39586665	Effects of antibiotic to ACLF	CLONE	METAGENOMIC	PCR
M14M_1	M14M_1	SAMN39586666	Effects of antibiotic to ACLF	CLONE	METAGENOMIC	PCR
M14M_2	M14M_2	SAMN39586667	Effects of antibiotic to ACLF	CLONE	METAGENOMIC	PCR
M16M_1	M16M_1	SAMN39586668	Effects of antibiotic to ACLF	CLONE	METAGENOMIC	PCR
M16M_2	M16M_2	SAMN39586669	Effects of antibiotic to ACLF	CLONE	METAGENOMIC	PCR
M19M_1	M19M_1	SAMN39586670	Effects of antibiotic to ACLF	CLONE	METAGENOMIC	PCR
M19M_2	M19M_2	SAMN39586671	Effects of antibiotic to ACLF	CLONE	METAGENOMIC	PCR
M21M_1	M21M_1	SAMN39586672	Effects of antibiotic to ACLF	CLONE	METAGENOMIC	PCR
M21M_2	M21M_2	SAMN39586673	Effects of antibiotic to ACLF	CLONE	METAGENOMIC	PCR
M23M_1	M23M_1	SAMN39586674	Effects of antibiotic to ACLF	CLONE	METAGENOMIC	PCR
M23M_2	M23M_2	SAMN39586675	Effects of antibiotic to ACLF	CLONE	METAGENOMIC	PCR
M24M_1	M24M_1	SAMN39586676	Effects of antibiotic to ACLF	CLONE	METAGENOMIC	PCR
M24M_2	M24M_2	SAMN39586677	Effects of antibiotic to ACLF	CLONE	METAGENOMIC	PCR
M26M_1	M26M_1	SAMN39586678	Effects of antibiotic to ACLF	CLONE	METAGENOMIC	PCR
M26M_2	M26M_2	SAMN39586679	Effects of antibiotic to ACLF	CLONE	METAGENOMIC	PCR
M27M_1	M27M_1	SAMN39586680	Effects of antibiotic to ACLF	CLONE	METAGENOMIC	PCR
M27M_2	M27M_2	SAMN39586681	Effects of antibiotic to ACLF	CLONE	METAGENOMIC	PCR
M28M_1	M28M_1	SAMN39586682	Effects of antibiotic to ACLF	CLONE	METAGENOMIC	PCR
M28M_2	M28M_2	SAMN39586683	Effects of antibiotic to ACLF	CLONE	METAGENOMIC	PCR
M29M_1	M29M_1	SAMN39586684	Effects of antibiotic to ACLF	CLONE	METAGENOMIC	PCR
M29M_2	M29M_2	SAMN39586685	Effects of antibiotic to ACLF	CLONE	METAGENOMIC	PCR
M32M_1	M32M_1	SAMN39586686	Effects of antibiotic to ACLF	CLONE	METAGENOMIC	PCR
M32M_2	M32M_2	SAMN39586687	Effects of antibiotic to ACLF	CLONE	METAGENOMIC	PCR

M34M_1	M34M_1	SAMN39586688	Effects of antibiotic to ACLF	CLONE	METAGENOMIC	PCR
M34M_2	M34M_2	SAMN39586689	Effects of antibiotic to ACLF	CLONE	METAGENOMIC	PCR
M36M_1	M36M_1	SAMN39586690	Effects of antibiotic to ACLF	CLONE	METAGENOMIC	PCR
M36M_2	M36M_2	SAMN39586691	Effects of antibiotic to ACLF	CLONE	METAGENOMIC	PCR
M38M_1	M38M_1	SAMN39586692	Effects of antibiotic to ACLF	CLONE	METAGENOMIC	PCR
M38M_2	M38M_2	SAMN39586693	Effects of antibiotic to ACLF	CLONE	METAGENOMIC	PCR
M41M_1	M41M_1	SAMN39586694	Effects of antibiotic to ACLF	CLONE	METAGENOMIC	PCR
M41M_2	M41M_2	SAMN39586695	Effects of antibiotic to ACLF	CLONE	METAGENOMIC	PCR
M56M_1	M56M_1	SAMN39586696	Effects of antibiotic to ACLF	CLONE	METAGENOMIC	PCR
M56M_2	M56M_2	SAMN39586697	Effects of antibiotic to ACLF	CLONE	METAGENOMIC	PCR
M60M_1	M60M_1	SAMN39586700	Effects of antibiotic to ACLF	CLONE	METAGENOMIC	PCR
M60M_2	M60M_2	SAMN39586699	Effects of antibiotic to ACLF	CLONE	METAGENOMIC	PCR
M62M_1	M62M_1	SAMN39586700	Effects of antibiotic to ACLF	CLONE	METAGENOMIC	PCR
M62M_2	M62M_2	SAMN39586701	Effects of antibiotic to ACLF	CLONE	METAGENOMIC	PCR
M63M_1	M63M_1	SAMN39586702	Effects of antibiotic to ACLF	CLONE	METAGENOMIC	PCR
M63M_2	M63M_2	SAMN39586703	Effects of antibiotic to ACLF	CLONE	METAGENOMIC	PCR
M65M_1	M65M_1	SAMN39586704	Effects of antibiotic to ACLF	CLONE	METAGENOMIC	PCR
M65M_2	M65M_2	SAMN39586705	Effects of antibiotic to ACLF	CLONE	METAGENOMIC	PCR
M68M_1	M68M_1	SAMN39586706	Effects of antibiotic to ACLF	CLONE	METAGENOMIC	PCR
M68M_2	M68M_2	SAMN39586707	Effects of antibiotic to ACLF	CLONE	METAGENOMIC	PCR
M71M_1	M71M_1	SAMN39586708	Effects of antibiotic to ACLF	CLONE	METAGENOMIC	PCR
M71M_2	M71M_2	SAMN39586709	Effects of antibiotic to ACLF	CLONE	METAGENOMIC	PCR
M72M_1	M72M_1	SAMN39586710	Effects of antibiotic to ACLF	CLONE	METAGENOMIC	PCR
M72M_2	M72M_2	SAMN39586711	Effects of antibiotic to ACLF	CLONE	METAGENOMIC	PCR
M78M_1	M78M_1	SAMN39586712	Effects of antibiotic to ACLF	CLONE	METAGENOMIC	PCR
M78M_2	M78M_2	SAMN39586713	Effects of antibiotic to ACLF	CLONE	METAGENOMIC	PCR
M80M_1	M80M_1	SAMN39586714	Effects of antibiotic to ACLF	CLONE	METAGENOMIC	PCR
M80M_2	M80M_2	SAMN39586715	Effects of antibiotic to ACLF	CLONE	METAGENOMIC	PCR
M81M_1	M81M_1	SAMN39586716	Effects of antibiotic to ACLF	CLONE	METAGENOMIC	PCR
M81M_2	M81M_2	SAMN39586717	Effects of antibiotic to ACLF	CLONE	METAGENOMIC	PCR
M83M_1	M83M_1	SAMN39586718	Effects of antibiotic to ACLF	CLONE	METAGENOMIC	PCR
M83M_2	M83M_2	SAMN39586719	Effects of antibiotic to ACLF	CLONE	METAGENOMIC	PCR
M85M_1	M85M_1	SAMN39586720	Effects of antibiotic to ACLF	CLONE	METAGENOMIC	PCR
M85M_2	M85M_2	SAMN39586721	Effects of antibiotic to ACLF	CLONE	METAGENOMIC	PCR
M86M_1	M86M_1	SAMN39586722	Effects of antibiotic to ACLF	CLONE	METAGENOMIC	PCR
M86M_2	M86M_2	SAMN39586723	Effects of antibiotic to ACLF	CLONE	METAGENOMIC	PCR
M87M_1	M87M_1	SAMN39586724	Effects of antibiotic to ACLF	CLONE	METAGENOMIC	PCR
M87M_2	M87M_2	SAMN39586725	Effects of antibiotic to ACLF	CLONE	METAGENOMIC	PCR
M88M_1	M88M_1	SAMN39586726	Effects of antibiotic to ACLF	CLONE	METAGENOMIC	PCR
M88M_2	M88M_2	SAMN39586727	Effects of antibiotic to ACLF	CLONE	METAGENOMIC	PCR
M89M_1	M89M_1	SAMN39586728	Effects of antibiotic to ACLF	CLONE	METAGENOMIC	PCR
M89M_2	M89M_2	SAMN39586733	Effects of antibiotic to ACLF	CLONE	METAGENOMIC	PCR

M91M_1	M91M_1	SAMN39586730	Effects of antibiotic to ACLF	CLONE	METAGENOMIC	PCR
M91M_2	M91M_2	SAMN39586733	Effects of antibiotic to ACLF	CLONE	METAGENOMIC	PCR
M92M_1	M92M_1	SAMN39586732	Effects of antibiotic to ACLF	CLONE	METAGENOMIC	PCR
M92M_2	M92M_2	SAMN39586733	Effects of antibiotic to ACLF	CLONE	METAGENOMIC	PCR
M93M_1	M93M_1	SAMN39586734	Effects of antibiotic to ACLF	CLONE	METAGENOMIC	PCR
M93M_2	M93M_2	SAMN39586735	Effects of antibiotic to ACLF	CLONE	METAGENOMIC	PCR
M94M_1	M94M_1	SAMN39586736	Effects of antibiotic to ACLF	CLONE	METAGENOMIC	PCR
M94M_2	M94M_2	SAMN39586737	Effects of antibiotic to ACLF	CLONE	METAGENOMIC	PCR
M98M_1	M98M_1	SAMN39586738	Effects of antibiotic to ACLF	CLONE	METAGENOMIC	PCR
M98M_2	M98M_2	SAMN39586739	Effects of antibiotic to ACLF	CLONE	METAGENOMIC	pCR
MC1M_1	MC1M_1	SAMN39586740	Effects of antibiotic to ACLF	CLONE	METAGENOMIC	PCR
MC1M_2	MC1M_2	SAMN39586741	Effects of antibiotic to ACLF	CLONE	METAGENOMIC	PCR
MC2M_1	MC2M_1	SAMN39586742	Effects of antibiotic to ACLF	CLONE	METAGENOMIC	PCR
MC2M_2	MC2M_2	SAMN39586743	Effects of antibiotic to ACLF	CLONE	METAGENOMIC	PCR
MC3M_1	MC3M_1	SAMN39586744	Effects of antibiotic to ACLF	CLONE	METAGENOMIC	PCR
MC3M_2	MC3M_2	SAMN39586745	Effects of antibiotic to ACLF	CLONE	METAGENOMIC	PCR
MC5M_1	MC5M_1	SAMN39586746	Effects of antibiotic to ACLF	CLONE	METAGENOMIC	PCR
MC5M_2	MC5M_2	SAMN39586747	Effects of antibiotic to ACLF	CLONE	METAGENOMIC	PCR
MC96M_:	MC96M_1	SAMN39586748	Effects of antibiotic to ACLF	CLONE	METAGENOMIC	PCR
MC96M_:	MC96M_2	SAMN39586749	Effects of antibiotic to ACLF	CLONE	METAGENOMIC	PCR
MC97M_:	MC97M_1	SAMN39586750	Effects of antibiotic to ACLF	CLONE	METAGENOMIC	PCR
MC97M_:	MC97M_2	SAMN39586751	Effects of antibiotic to ACLF	CLONE	METAGENOMIC	PCR

library_layout	platform	instrument_model	design_description	filetype	filename	filename2	filename3
single	ILLUMINA	Illumina MiSeq	faeces sample	fastq	M1M_1.fq		
single	ILLUMINA	Illumina MiSeq	faeces sample	fastq	M1M_2.fq		
single	ILLUMINA	Illumina MiSeq	faeces sample	fastq	M2M_1.fq		
single	ILLUMINA	Illumina MiSeq	faeces sample	fastq	M2M_2.fq		
single	ILLUMINA	Illumina MiSeq	faeces sample	fastq	M3M_1.fq		
single	ILLUMINA	Illumina MiSeq	faeces sample	fastq	M3M_2.fq		
single	ILLUMINA	Illumina MiSeq	faeces sample	fastq	M4M_1.fq		
single	ILLUMINA	Illumina MiSeq	faeces sample	fastq	M4M_2.fq		
single	ILLUMINA	Illumina MiSeq	faeces sample	fastq	M7M_1.fq		
single	ILLUMINA	Illumina MiSeq	faeces sample	fastq	M7M_2.fq		
single	ILLUMINA	Illumina MiSeq	faeces sample	fastq	M8M_1.fq		
single	ILLUMINA	Illumina MiSeq	faeces sample	fastq	M8M_2.fq		
single	ILLUMINA	Illumina MiSeq	faeces sample	fastq	M11M_1.fq		
single	ILLUMINA	Illumina MiSeq	faeces sample	fastq	M11M_2.fq		
single	ILLUMINA	Illumina MiSeq	faeces sample	fastq	M12M_1.fq		
single	ILLUMINA	Illumina MiSeq	faeces sample	fastq	M12M_2.fq		
single	ILLUMINA	Illumina MiSeq	faeces sample	fastq	M14M_1.fq		
single	ILLUMINA	Illumina MiSeq	faeces sample	fastq	M14M_2.fq		
single	ILLUMINA	Illumina MiSeq	faeces sample	fastq	M16M_1.fq		
single	ILLUMINA	Illumina MiSeq	faeces sample	fastq	M16M_2.fq		
single	ILLUMINA	Illumina MiSeq	faeces sample	fastq	M19M_1.fq		
single	ILLUMINA	Illumina MiSeq	faeces sample	fastq	M19M_2.fq		
single	ILLUMINA	Illumina MiSeq	faeces sample	fastq	M21M_1.fq		
single	ILLUMINA	Illumina MiSeq	faeces sample	fastq	M21M_2.fq		
single	ILLUMINA	Illumina MiSeq	faeces sample	fastq	M23M_1.fq		
single	ILLUMINA	Illumina MiSeq	faeces sample	fastq	M23M_2.fq		
single	ILLUMINA	Illumina MiSeq	faeces sample	fastq	M24M_1.fq		
single	ILLUMINA	Illumina MiSeq	faeces sample	fastq	M24M_2.fq		
single	ILLUMINA	Illumina MiSeq	faeces sample	fastq	M26M_1.fq		
single	ILLUMINA	Illumina MiSeq	faeces sample	fastq	M26M_2.fq		
single	ILLUMINA	Illumina MiSeq	faeces sample	fastq	M27M_1.fq		
single	ILLUMINA	Illumina MiSeq	faeces sample	fastq	M27M_2.fq		
single	ILLUMINA	Illumina MiSeq	faeces sample	fastq	M28M_1.fq		
single	ILLUMINA	Illumina MiSeq	faeces sample	fastq	M28M_2.fq		
single	ILLUMINA	Illumina MiSeq	faeces sample	fastq	M29M_1.fq		
single	ILLUMINA	Illumina MiSeq	faeces sample	fastq	M29M_2.fq		
single	ILLUMINA	Illumina MiSeq	faeces sample	fastq	M32M_1.fq		
single	ILLUMINA	Illumina MiSeq	faeces sample	fastq	M32M_2.fq		

single	ILLUMINA	Illumina MiSeq	faeces sample	fastq	M34M_1.fq
single	ILLUMINA	Illumina MiSeq	faeces sample	fastq	M34M_2.fq
single	ILLUMINA	Illumina MiSeq	faeces sample	fastq	M36M_1.fq
single	ILLUMINA	Illumina MiSeq	faeces sample	fastq	M36M_2.fq
single	ILLUMINA	Illumina MiSeq	faeces sample	fastq	M38M_1.fq
single	ILLUMINA	Illumina MiSeq	faeces sample	fastq	M38M_2.fq
single	ILLUMINA	Illumina MiSeq	faeces sample	fastq	M41M_1.fq
single	ILLUMINA	Illumina MiSeq	faeces sample	fastq	M41M_2.fq
single	ILLUMINA	Illumina MiSeq	faeces sample	fastq	M56M_1.fq
single	ILLUMINA	Illumina MiSeq	faeces sample	fastq	M56M_2.fq
single	ILLUMINA	Illumina MiSeq	faeces sample	fastq	M60M_1.fq
single	ILLUMINA	Illumina MiSeq	faeces sample	fastq	M60M_2.fq
single	ILLUMINA	Illumina MiSeq	faeces sample	fastq	M62M_1.fq
single	ILLUMINA	Illumina MiSeq	faeces sample	fastq	M62M_2.fq
single	ILLUMINA	Illumina MiSeq	faeces sample	fastq	M63M_1.fq
single	ILLUMINA	Illumina MiSeq	faeces sample	fastq	M63M_2.fq
single	ILLUMINA	Illumina MiSeq	faeces sample	fastq	M65M_1.fq
single	ILLUMINA	Illumina MiSeq	faeces sample	fastq	M65M_2.fq
single	ILLUMINA	Illumina MiSeq	faeces sample	fastq	M68M_1.fq
single	ILLUMINA	Illumina MiSeq	faeces sample	fastq	M68M_2.fq
single	ILLUMINA	Illumina MiSeq	faeces sample	fastq	M71M_1.fq
single	ILLUMINA	Illumina MiSeq	faeces sample	fastq	M71M_2.fq
single	ILLUMINA	Illumina MiSeq	faeces sample	fastq	M72M_1.fq
single	ILLUMINA	Illumina MiSeq	faeces sample	fastq	M72M_2.fq
single	ILLUMINA	Illumina MiSeq	faeces sample	fastq	M78M_1.fq
single	ILLUMINA	Illumina MiSeq	faeces sample	fastq	M78M_2.fq
single	ILLUMINA	Illumina MiSeq	faeces sample	fastq	M80M_1.fq
single	ILLUMINA	Illumina MiSeq	faeces sample	fastq	M80M_2.fq
single	ILLUMINA	Illumina MiSeq	faeces sample	fastq	M81M_1.fq
single	ILLUMINA	Illumina MiSeq	faeces sample	fastq	M81M_2.fq
single	ILLUMINA	Illumina MiSeq	faeces sample	fastq	M83M_1.fq
single	ILLUMINA	Illumina MiSeq	faeces sample	fastq	M83M_2.fq
single	ILLUMINA	Illumina MiSeq	faeces sample	fastq	M85M_1.fq
single	ILLUMINA	Illumina MiSeq	faeces sample	fastq	M85M_2.fq
single	ILLUMINA	Illumina MiSeq	faeces sample	fastq	M86M_1.fq
single	ILLUMINA	Illumina MiSeq	faeces sample	fastq	M86M_2.fq
single	ILLUMINA	Illumina MiSeq	faeces sample	fastq	M87M_1.fq
single	ILLUMINA	Illumina MiSeq	faeces sample	fastq	M87M_2.fq
single	ILLUMINA	Illumina MiSeq	faeces sample	fastq	M88M_1.fq
single	ILLUMINA	Illumina MiSeq	faeces sample	fastq	M88M_2.fq
single	ILLUMINA	Illumina MiSeq	faeces sample	fastq	M89M_1.fq
single	ILLUMINA	Illumina MiSeq	faeces sample	fastq	M89M_2.fq

single	ILLUMINA	Illumina MiSeq	faeces sample	fastq	M91M_1.fq
single	ILLUMINA	Illumina MiSeq	faeces sample	fastq	M91M_2.fq
single	ILLUMINA	Illumina MiSeq	faeces sample	fastq	M92M_1.fq
single	ILLUMINA	Illumina MiSeq	faeces sample	fastq	M92M_2.fq
single	ILLUMINA	Illumina MiSeq	faeces sample	fastq	M93M_1.fq
single	ILLUMINA	Illumina MiSeq	faeces sample	fastq	M93M_2.fq
single	ILLUMINA	Illumina MiSeq	faeces sample	fastq	M94M_1.fq
single	ILLUMINA	Illumina MiSeq	faeces sample	fastq	M94M_2.fq
single	ILLUMINA	Illumina MiSeq	faeces sample	fastq	M98M_1.fq
single	ILLUMINA	Illumina MiSeq	faeces sample	fastq	M98M_2.fq
single	ILLUMINA	Illumina MiSeq	faeces sample	fastq	MC1M_1.fq
single	ILLUMINA	Illumina MiSeq	faeces sample	fastq	MC1M_2.fq
single	ILLUMINA	Illumina MiSeq	faeces sample	fastq	MC2M_1.fq
single	ILLUMINA	Illumina MiSeq	faeces sample	fastq	MC2M_2.fq
single	ILLUMINA	Illumina MiSeq	faeces sample	fastq	MC3M_1.fq
single	ILLUMINA	Illumina MiSeq	faeces sample	fastq	MC3M_2.fq
single	ILLUMINA	Illumina MiSeq	faeces sample	fastq	MC5M_1.fq
single	ILLUMINA	Illumina MiSeq	faeces sample	fastq	MC5M_2.fq
single	ILLUMINA	Illumina MiSeq	faeces sample	fastq	MC96M_1.fq
single	ILLUMINA	Illumina MiSeq	faeces sample	fastq	MC96M_2.fq
single	ILLUMINA	Illumina MiSeq	faeces sample	fastq	MC97M_1.fq
single	ILLUMINA	Illumina MiSeq	faeces sample	fastq	MC97M_2.fq
